# 2,3-Di­hydro-1*H*-cyclo­penta­[*b*]naphthalene-4,9-dione

**DOI:** 10.1107/S241431462100167X

**Published:** 2021-02-16

**Authors:** Sambasivarao Kotha, Ambareen Fatma, Saima Ansari

**Affiliations:** a Department of Chemistry, Indian Institute of Technology Bombay, Powai, Mumbai - 400076, India; University of Aberdeen, Scotland

**Keywords:** naphtho­quinone, retro Diels–Alder reaction, cyclo­reversion, planarity, crystal structure

## Abstract

The title compound, C_13_H_10_O_2_, crystallizes with two almost planar mol­ecules in the asymmetric unit. In the crystal, slipped π–π stacking inter­actions help to establish the packing with the shortest centroid–centroid separation being 3.8195 (18) Å.

## Abstract 2

The crystal structure of the title penta­cyclo-substituted naphtho­quinone, C_13_H_10_O_2_ is described.

## Graphical abstract

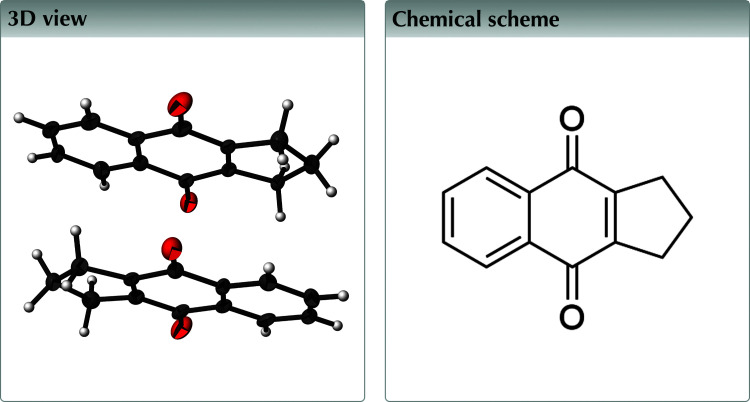

## Structure description

Several kinds of naphtho­quinone ring-containing compounds have been encountered in nature that are known to be biologically important mol­ecules (Qiu *et al.*, 2018 ▸). Naphtho­quinones are also important constituents of a variety of quinone-based dyes upon ring fusion with heterocyclic system (Katritzky *et al.*, 1988 ▸). We now describe the synthesis and structure of the title compound, **2**.

**Figure 1:**
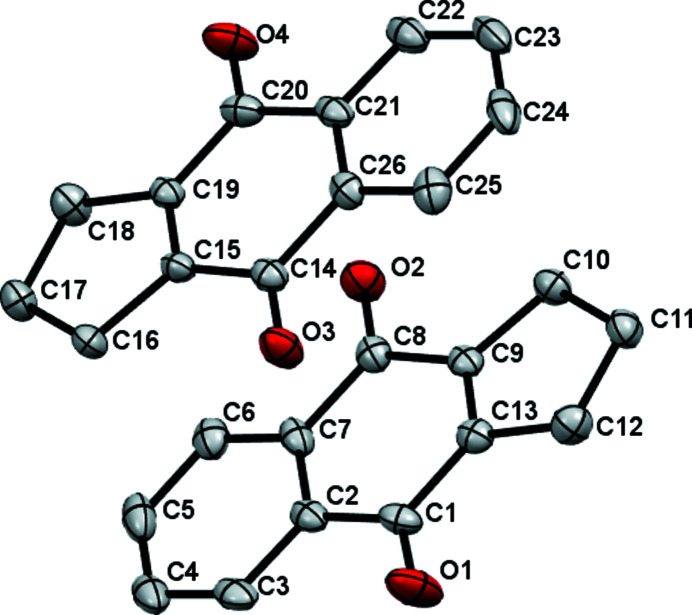
The mol­ecular structure of the title compound with displacement ellipsoids drawn at the 50% level while H atoms are omitted for clarity.

Compound **2** crystallizes with two almost planar mol­ecules in the asymmetric unit (Fig. 1 ▸). For the C1-mol­ecule, the C=O bonds (C1=O1 and C8=O2) are almost the same length [1.221 (4) Å and 1.225 (4) Å, respectively]. The C9=C13 double bond connected with the fused cyclo­pentane ring is shorter [1.343 (5) Å] than the equivalent bond found in 2-hy­droxy-3-(2-methyl­prop-1-en-1-yl)naphthalene 1,4-dione (Alcantara Emiliano *et al.*, 2016 ▸; Cambridge Structural Database refcode XAHPAA) [1.361 (3) Å] due, presumably, to ring strain (Fig. 1 ▸). A difference of 2° is found between the angles C1—C13—C9 [123.2 (3)°] and C8—C9—C13 [121.7 (3)°] in the title compound whereas in XAHPAA this difference is about 4° owing to the different substituents. The second C14 mol­ecule, with a similar geometry to the C1 mol­ecule, completes the asymmetric unit of the title compound.

**Figure 2:**
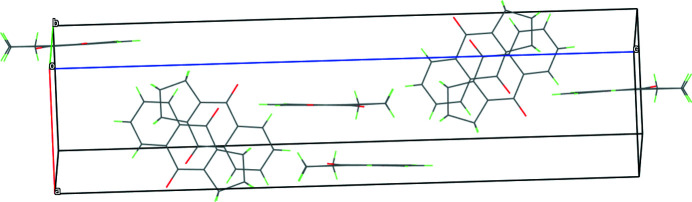
The crystal packing viewed along the *b*-axis direction.

The packing is represented in Fig. 2 ▸. There is no direct evidence of any intra- or inter­molecular hydrogen bonding in the system. Adjacent mol­ecules are stacked in an angular (shifted) orientation and several slipped aromatic π–π stacking inter­actions help to establish the packing, the shortest centroid–centroid separation being 3.8195 (18) Å (slippage = 1.722 Å) for the C1/C2/C7–C9/C13 and C14/C15/C19–C21/C26 rings.

## Synthesis and crystallization

**Figure 3:**
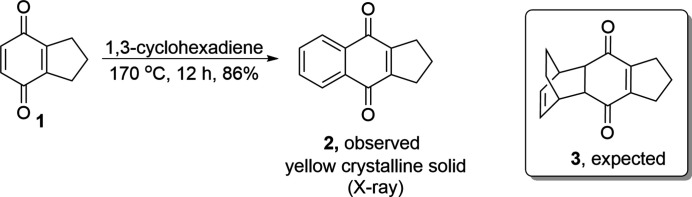
Synthesis scheme for the title compound.

As part of our studies of Diels–Alder chemistry, we explored the DA reaction between 1,3-cyclohexa­diene and the quinone derivative **1** at 170°C in a sealed tube for 12 h (Fig. 3 ▸). To our surprise, instead of forming the expected DA adduct **3**, the aromatized title compound was achieved in an excellent yield (86%) as a yellow crystalline solid. We suggest that initially the expected [4 + 2] cyclo­addition reaction happened, but at high temperature the cyclo­adduct underwent cyclo­reversion (retro-DA reaction), resulting in the elimination of a volatile ethyl­ene mol­ecule and aromatization. The compound was recrystallized from mixed solvents of petroleum ether and ethyl acetate (4:1) in the refrigerator. Yellow crystalline solid; ^1^H NMR (400 MHz, CDCl_3_): *δ* = 8.06 (*J* = 5.72, 3.37 Hz, 2H), 7.70 (*dd*, *J* = 5.65, 3.31 Hz, 2H), 2.94 (*t*, *J* = 7.75 Hz, 4H), 2.09 (*m*, 2H) p.p.m.; ^13^C NMR (100 MHz, CDCl_3_): *δ* = 183.9, 151.2, 133.3, 133.1, 126.1, 31.1, 21.5 p.p.m.. (Clausen *et al.*, 2001 ▸; Franck *et al.*, 1985 ▸).

## Refinement

Crystal data, data collection and structure refinement details are summarized in Table 1 ▸.

## Supplementary Material

Crystal structure: contains datablock(s) global, I. DOI: 10.1107/S241431462100167X/hb4372sup1.cif

Structure factors: contains datablock(s) I. DOI: 10.1107/S241431462100167X/hb4372Isup2.hkl

Supporting information file. DOI: 10.1107/S241431462100167X/hb4372Isup3.cml

CCDC reference: 2046748

Additional supporting information:  crystallographic information; 3D view; checkCIF report

## Figures and Tables

**Table 1:** Experimental details

Crystal data	
Chemical formula	C_13_H_10_O_2_
*M*_r_	198.22
Crystal system, space group	Tetragonal, *P*4_3_
Temperature (K)	150
*a*, *c* (Å)	7.4781 (1), 33.0219 (10)
*V* (Å^3^)	1846.65 (7)
*Z*	8
Radiation type	Mo *K*α
μ (mm^−1^)	0.10
Crystal size (mm)	0.21 × 0.13 × 0.04

Data collection	
Diffractometer	Rigaku Oxford Diffraction CCD
Absorption correction	Multi-scan (*CrysAlis PRO*; Rigaku OD, 2018 ▸)
*T*_min_, *T*_max_	0.636, 1.000
No. of measured, independent and observed [*I* ≥ 2u(*I*)] reflections	46308, 3230, 2851
*R*_int_	0.150
(sin θ/λ)_max_ (Å^−1^)	0.594

Refinement	
*R*[*F* ^2^ > 2σ(*F* ^2^)], *wR*(*F* ^2^), *S*	0.060, 0.182, 1.05
No. of reflections	3230
No. of parameters	271
No. of restraints	1
H-atom treatment	H-atom parameters constrained
Δρ_max_, Δρ_min_ (e Å^−3^)	0.35, −0.32
Absolute structure	Flack (1983 ▸)
Absolute structure parameter	1.0 (16)

Computer programs: *CrysAlis PRO* (Rigaku OD, 2018 ▸), *olex2.solve* (Bourhis *et al.*, 2015 ▸), *olex2.refine* (Bourhis *et al.*, 2015 ▸) and *OLEX2* (Dolomanov *et al.*, 2009 ▸).

## full crystallographic data

### Crystal data

**Table:**

C_13_H_10_O_2_	*D*_x_ = 1.426 Mg m^−^^3^
*M**_r_* = 198.22	Mo *K*α radiation, λ = 0.71073 Å
Tetragonal, *P*4_3_	Cell parameters from 16230 reflections
*a* = 7.4781 (1) Å	θ = 2.5–30.9°
*c* = 33.0219 (10) Å	µ = 0.10 mm^−^^1^
*V* = 1846.65 (7) Å^3^	*T* = 150 K
*Z* = 8	Block, colourless
*F*(000) = 832.447	0.21 × 0.13 × 0.04 mm

### Data collection

**Table:**

Rigaku Oxford Diffraction CCD diffractometer	3230 independent reflections
Radiation source: fine-focus sealed X-ray tube, Enhance (Mo) X-ray Source	2851 reflections with *I*≥ 2u(*I*)
Graphite monochromator	*R*_int_ = 0.150
ω scans	θ_max_ = 25.0°, θ_min_ = 2.5°
Absorption correction: multi-scan (CrysAlisPro; Rigaku OD, 2018)	*h* = −10→10
*T*_min_ = 0.636, *T*_max_ = 1.000	*k* = −10→10
46308 measured reflections	*l* = −46→47

### Refinement

**Table:**

Refinement on *F*^2^	Primary atom site location: iterative
Least-squares matrix: full	H-atom parameters constrained
*R*[*F*^2^ > 2σ(*F*^2^)] = 0.060	*w* = 1/[σ^2^(*F*_o_^2^) + (0.1209*P*)^2^ + 0.4577*P*] where *P* = (*F*_o_^2^ + 2*F*_c_^2^)/3
*wR*(*F*^2^) = 0.182	(Δ/σ)_max_ = 0.0002
*S* = 1.05	Δρ_max_ = 0.35 e Å^−^^3^
3230 reflections	Δρ_min_ = −0.31 e Å^−^^3^
271 parameters	Absolute structure: Flack (1983)
1 restraint	Absolute structure parameter: 1.0 (16)
34 constraints	

### Fractional atomic coordinates and isotropic or equivalent isotropic displacement parameters (Å^2^)

**Table:**

	*x*	*y*	*z*	*U*_iso_*/*U*_eq_	
O2	−0.1998 (3)	−1.0652 (3)	−0.53158 (8)	0.0313 (6)	
O3	−0.4644 (3)	−0.5372 (3)	−0.46901 (7)	0.0314 (6)	
O1	0.0178 (4)	−0.5172 (4)	−0.43869 (8)	0.0414 (7)	
O4	−0.6811 (4)	−1.0853 (4)	−0.56192 (8)	0.0395 (7)	
C13	−0.0343 (4)	−0.6351 (4)	−0.50334 (11)	0.0217 (8)	
C8	−0.1458 (4)	−0.9450 (4)	−0.50952 (11)	0.0223 (8)	
C15	−0.5855 (4)	−0.8269 (5)	−0.47358 (9)	0.0199 (7)	
C7	−0.1437 (4)	−0.9603 (4)	−0.46489 (11)	0.0233 (8)	
C2	−0.0910 (4)	−0.8149 (5)	−0.44071 (10)	0.0229 (8)	
C1	−0.0324 (4)	−0.6440 (4)	−0.45901 (11)	0.0239 (8)	
C16	−0.5999 (4)	−0.8685 (4)	−0.42918 (9)	0.0232 (7)	
H16a	−0.4821 (4)	−0.8576 (4)	−0.41571 (9)	0.0278 (9)*	
H16b	−0.6857 (4)	−0.7871 (4)	−0.41571 (9)	0.0278 (9)*	
C6	−0.1933 (5)	−1.1217 (5)	−0.44638 (11)	0.0276 (8)	
H6	−0.2294 (5)	−1.2205 (5)	−0.46249 (11)	0.0331 (10)*	
C9	−0.0811 (4)	−0.7738 (4)	−0.52694 (9)	0.0202 (7)	
C26	−0.5235 (4)	−0.6405 (4)	−0.53587 (11)	0.0218 (8)	
C18	−0.6846 (5)	−1.1272 (5)	−0.47248 (10)	0.0272 (8)	
H18a	−0.6030 (5)	−1.2287 (5)	−0.47791 (10)	0.0327 (10)*	
H18b	−0.8087 (5)	−1.1644 (5)	−0.47857 (10)	0.0327 (10)*	
C17	−0.6672 (5)	−1.0628 (4)	−0.42807 (10)	0.0257 (8)	
H17a	−0.5815 (5)	−1.1392 (4)	−0.41314 (10)	0.0308 (9)*	
H17b	−0.7846 (5)	−1.0690 (4)	−0.41429 (10)	0.0308 (9)*	
C14	−0.5197 (4)	−0.6577 (4)	−0.49071 (10)	0.0219 (7)	
C20	−0.6329 (4)	−0.9584 (4)	−0.54108 (11)	0.0260 (8)	
C10	−0.0680 (4)	−0.7337 (4)	−0.57106 (10)	0.0240 (7)	
H10a	0.0172 (4)	−0.8154 (4)	−0.58461 (10)	0.0288 (9)*	
H10b	−0.1862 (4)	−0.7440 (4)	−0.58437 (10)	0.0288 (9)*	
C21	−0.5758 (4)	−0.7871 (5)	−0.56003 (10)	0.0242 (8)	
C25	−0.4721 (5)	−0.4794 (5)	−0.55371 (12)	0.0293 (8)	
H25	−0.4349 (5)	−0.3815 (5)	−0.53744 (12)	0.0352 (10)*	
C4	−0.1399 (4)	−0.9941 (6)	−0.38067 (12)	0.0334 (9)	
H4	−0.1397 (4)	−1.0054 (6)	−0.35201 (12)	0.0401 (11)*	
C24	−0.4762 (5)	−0.4644 (5)	−0.59580 (11)	0.0337 (9)	
H24	−0.4440 (5)	−0.3544 (5)	−0.60823 (11)	0.0404 (11)*	
C19	−0.6330 (4)	−0.9670 (4)	−0.49681 (11)	0.0197 (8)	
C11	0.0008 (5)	−0.5378 (4)	−0.57204 (10)	0.0265 (8)	
H11a	0.1181 (5)	−0.5317 (4)	−0.58585 (10)	0.0318 (9)*	
H11b	−0.0847 (5)	−0.4605 (4)	−0.58681 (10)	0.0318 (9)*	
C12	0.0185 (5)	−0.4755 (5)	−0.52765 (11)	0.0282 (8)	
H12a	−0.0620 (5)	−0.3733 (5)	−0.52209 (11)	0.0339 (10)*	
H12b	0.1430 (5)	−0.4396 (5)	−0.52153 (11)	0.0339 (10)*	
C22	−0.5754 (5)	−0.7690 (5)	−0.60195 (10)	0.0306 (8)	
H22	−0.6086 (5)	−0.8673 (5)	−0.61857 (10)	0.0368 (10)*	
C23	−0.5266 (4)	−0.6074 (6)	−0.61955 (11)	0.0350 (9)	
H23	−0.5279 (4)	−0.5954 (6)	−0.64819 (11)	0.0420 (11)*	
C3	−0.0899 (4)	−0.8328 (5)	−0.39846 (10)	0.0292 (8)	
H3	−0.0550 (4)	−0.7348 (5)	−0.38194 (10)	0.0351 (10)*	
C5	−0.1894 (5)	−1.1370 (5)	−0.40428 (11)	0.0342 (9)	
H5	−0.2213 (5)	−1.2470 (5)	−0.39185 (11)	0.0410 (11)*	

### Atomic displacement parameters (Å^2^)

**Table:**

	*U*^11^	*U*^22^	*U*^33^	*U*^12^	*U*^13^	*U*^23^
O2	0.0404 (14)	0.0252 (13)	0.0283 (13)	−0.0019 (11)	−0.0009 (11)	−0.0025 (10)
O3	0.0432 (15)	0.0280 (13)	0.0231 (13)	−0.0011 (11)	−0.0008 (11)	−0.0038 (11)
O1	0.0612 (18)	0.0350 (15)	0.0278 (15)	−0.0011 (12)	−0.0138 (13)	−0.0077 (13)
O4	0.0519 (17)	0.0388 (15)	0.0278 (14)	0.0009 (12)	−0.0114 (12)	−0.0089 (12)
C13	0.0200 (17)	0.0225 (19)	0.0226 (19)	0.0046 (13)	−0.0004 (12)	−0.0008 (12)
C8	0.0207 (16)	0.0237 (17)	0.0224 (18)	0.0048 (13)	0.0017 (13)	0.0018 (14)
C15	0.0193 (16)	0.0252 (17)	0.0152 (17)	0.0025 (13)	−0.0011 (13)	−0.0021 (13)
C7	0.0214 (18)	0.0285 (19)	0.0201 (18)	0.0087 (14)	0.0030 (12)	0.0069 (13)
C2	0.0180 (16)	0.0321 (19)	0.0186 (17)	0.0082 (13)	−0.0012 (13)	0.0000 (14)
C1	0.0257 (19)	0.0261 (19)	0.0198 (18)	0.0070 (14)	−0.0066 (12)	−0.0026 (13)
C16	0.0254 (17)	0.0268 (18)	0.0172 (17)	−0.0004 (13)	−0.0023 (14)	0.0036 (14)
C6	0.0210 (17)	0.032 (2)	0.0299 (19)	0.0049 (13)	0.0032 (14)	0.0059 (16)
C9	0.0165 (15)	0.0245 (17)	0.0198 (18)	0.0058 (12)	−0.0016 (12)	0.0016 (13)
C26	0.0207 (17)	0.0245 (18)	0.0202 (19)	0.0072 (13)	0.0032 (12)	−0.0001 (13)
C18	0.036 (2)	0.0204 (17)	0.0253 (19)	0.0014 (13)	0.0009 (15)	−0.0004 (14)
C17	0.0302 (18)	0.0264 (18)	0.0205 (17)	0.0016 (14)	0.0034 (14)	0.0011 (14)
C14	0.0215 (16)	0.0242 (17)	0.0200 (18)	0.0042 (13)	−0.0013 (12)	0.0002 (14)
C20	0.0250 (19)	0.031 (2)	0.022 (2)	0.0047 (14)	−0.0052 (13)	−0.0060 (14)
C10	0.0271 (18)	0.0243 (17)	0.0207 (17)	0.0020 (13)	0.0008 (14)	−0.0027 (14)
C21	0.0188 (17)	0.036 (2)	0.0173 (16)	0.0076 (14)	−0.0020 (13)	−0.0008 (14)
C25	0.0279 (19)	0.0276 (19)	0.032 (2)	0.0069 (14)	0.0060 (15)	0.0049 (16)
C4	0.0206 (18)	0.056 (2)	0.0234 (19)	0.0075 (15)	0.0009 (13)	0.0121 (17)
C24	0.0265 (19)	0.046 (2)	0.028 (2)	0.0086 (16)	0.0044 (15)	0.0196 (17)
C19	0.0178 (16)	0.0238 (19)	0.0176 (18)	0.0040 (13)	−0.0009 (12)	0.0010 (12)
C11	0.0280 (18)	0.0280 (18)	0.0235 (18)	0.0000 (13)	0.0009 (14)	0.0057 (15)
C12	0.0293 (18)	0.0250 (17)	0.030 (2)	−0.0005 (13)	0.0003 (15)	−0.0028 (15)
C22	0.0252 (18)	0.047 (2)	0.0198 (17)	0.0102 (15)	0.0000 (14)	−0.0021 (16)
C23	0.0242 (19)	0.064 (3)	0.0165 (18)	0.0111 (17)	0.0017 (13)	0.0057 (18)
C3	0.0209 (17)	0.044 (2)	0.0225 (18)	0.0096 (14)	−0.0060 (14)	−0.0002 (16)
C5	0.0239 (18)	0.046 (2)	0.033 (2)	0.0082 (15)	0.0094 (16)	0.0156 (18)

### Geometric parameters (Å, º)

**Table:**

O2—C8	1.225 (4)	C18—C17	1.549 (5)
O3—C14	1.223 (4)	C18—C19	1.493 (5)
O1—C1	1.221 (4)	C17—H17a	0.9900
O4—C20	1.226 (4)	C17—H17b	0.9900
C13—C1	1.465 (5)	C20—C21	1.488 (5)
C13—C9	1.343 (5)	C20—C19	1.463 (5)
C13—C12	1.492 (5)	C10—H10a	0.9900
C8—C7	1.478 (5)	C10—H10b	0.9900
C8—C9	1.485 (5)	C10—C11	1.553 (5)
C15—C16	1.503 (4)	C21—C22	1.391 (5)
C15—C14	1.471 (5)	C25—H25	0.9500
C15—C19	1.346 (5)	C25—C24	1.395 (5)
C7—C2	1.406 (5)	C4—H4	0.9500
C7—C6	1.403 (5)	C4—C3	1.393 (5)
C2—C1	1.480 (5)	C4—C5	1.373 (6)
C2—C3	1.402 (5)	C24—H24	0.9500
C16—H16a	0.9900	C24—C23	1.379 (6)
C16—H16b	0.9900	C11—H11a	0.9900
C16—C17	1.538 (5)	C11—H11b	0.9900
C6—H6	0.9500	C11—C12	1.544 (5)
C6—C5	1.395 (5)	C12—H12a	0.9900
C9—C10	1.491 (4)	C12—H12b	0.9900
C26—C14	1.497 (5)	C22—H22	0.9500
C26—C21	1.411 (5)	C22—C23	1.390 (5)
C26—C25	1.395 (5)	C23—H23	0.9500
C18—H18a	0.9900	C3—H3	0.9500
C18—H18b	0.9900	C5—H5	0.9500
			
C9—C13—C1	123.2 (3)	C21—C20—O4	121.0 (3)
C12—C13—C1	124.8 (3)	C19—C20—O4	121.8 (3)
C12—C13—C9	112.0 (3)	C19—C20—C21	117.3 (3)
C7—C8—O2	122.6 (3)	H10a—C10—C9	111.08 (18)
C9—C8—O2	120.6 (3)	H10b—C10—C9	111.08 (17)
C9—C8—C7	116.7 (3)	H10b—C10—H10a	109.0
C14—C15—C16	125.2 (3)	C11—C10—C9	103.4 (3)
C19—C15—C16	112.1 (3)	C11—C10—H10a	111.08 (18)
C19—C15—C14	122.6 (3)	C11—C10—H10b	111.08 (19)
C2—C7—C8	120.6 (3)	C20—C21—C26	120.7 (3)
C6—C7—C8	119.9 (3)	C22—C21—C26	119.1 (3)
C6—C7—C2	119.5 (3)	C22—C21—C20	120.2 (3)
C1—C2—C7	121.3 (3)	H25—C25—C26	120.5 (2)
C3—C2—C7	119.5 (3)	C24—C25—C26	119.0 (4)
C3—C2—C1	119.2 (3)	C24—C25—H25	120.5 (2)
C13—C1—O1	121.1 (3)	C3—C4—H4	119.8 (2)
C2—C1—O1	122.5 (3)	C5—C4—H4	119.8 (2)
C2—C1—C13	116.3 (3)	C5—C4—C3	120.4 (4)
H16a—C16—C15	110.96 (17)	H24—C24—C25	119.7 (2)
H16b—C16—C15	110.96 (17)	C23—C24—C25	120.7 (4)
H16b—C16—H16a	109.0	C23—C24—H24	119.7 (2)
C17—C16—C15	104.0 (3)	C18—C19—C15	112.7 (3)
C17—C16—H16a	110.96 (18)	C20—C19—C15	122.3 (3)
C17—C16—H16b	110.96 (18)	C20—C19—C18	125.0 (3)
H6—C6—C7	120.0 (2)	H11a—C11—C10	110.31 (18)
C5—C6—C7	119.9 (4)	H11b—C11—C10	110.31 (19)
C5—C6—H6	120.0 (2)	H11b—C11—H11a	108.6
C8—C9—C13	121.7 (3)	C12—C11—C10	107.1 (3)
C10—C9—C13	113.3 (3)	C12—C11—H11a	110.31 (17)
C10—C9—C8	125.0 (3)	C12—C11—H11b	110.31 (18)
C21—C26—C14	120.1 (3)	C11—C12—C13	104.3 (3)
C25—C26—C14	119.3 (3)	H12a—C12—C13	110.90 (19)
C25—C26—C21	120.6 (3)	H12a—C12—C11	110.90 (18)
H18b—C18—H18a	109.0	H12b—C12—C13	110.90 (18)
C17—C18—H18a	111.00 (19)	H12b—C12—C11	110.90 (17)
C17—C18—H18b	111.00 (18)	H12b—C12—H12a	108.9
C19—C18—H18a	111.00 (18)	H22—C22—C21	120.0 (2)
C19—C18—H18b	111.00 (18)	C23—C22—C21	120.1 (4)
C19—C18—C17	103.8 (3)	C23—C22—H22	120.0 (2)
C18—C17—C16	107.4 (3)	C22—C23—C24	120.6 (4)
H17a—C17—C16	110.24 (18)	H23—C23—C24	119.7 (2)
H17a—C17—C18	110.24 (19)	H23—C23—C22	119.7 (2)
H17b—C17—C16	110.24 (19)	C4—C3—C2	120.1 (4)
H17b—C17—C18	110.24 (19)	H3—C3—C2	120.0 (2)
H17b—C17—H17a	108.5	H3—C3—C4	120.0 (2)
C15—C14—O3	121.4 (3)	C4—C5—C6	120.5 (4)
C26—C14—O3	121.8 (3)	H5—C5—C6	119.7 (2)
C26—C14—C15	116.8 (3)	H5—C5—C4	119.7 (2)
			
O2—C8—C7—C2	−176.0 (3)	C8—C7—C2—C3	−179.9 (3)
O2—C8—C7—C6	4.5 (4)	C8—C7—C6—C5	179.3 (3)
O2—C8—C9—C13	174.8 (3)	C8—C9—C10—C11	178.4 (3)
O2—C8—C9—C10	−2.6 (4)	C15—C16—C17—C18	1.4 (3)
O3—C14—C15—C16	2.7 (4)	C15—C14—C26—C21	−4.6 (3)
O3—C14—C15—C19	−174.3 (3)	C15—C14—C26—C25	176.5 (3)
O3—C14—C26—C21	175.3 (3)	C15—C19—C18—C17	1.7 (3)
O3—C14—C26—C25	−3.7 (4)	C15—C19—C20—C21	0.6 (3)
O1—C1—C13—C9	177.1 (3)	C7—C2—C3—C4	0.3 (3)
O1—C1—C13—C12	−1.4 (4)	C7—C6—C5—C4	1.0 (4)
O1—C1—C2—C7	−178.3 (3)	C2—C3—C4—C5	0.4 (4)
O1—C1—C2—C3	0.2 (4)	C16—C17—C18—C19	−1.8 (3)
O4—C20—C21—C26	178.9 (3)	C6—C5—C4—C3	−1.1 (4)
O4—C20—C21—C22	−0.4 (4)	C9—C10—C11—C12	−1.4 (3)
O4—C20—C19—C15	−178.0 (3)	C26—C21—C20—C19	0.3 (3)
O4—C20—C19—C18	1.9 (4)	C26—C21—C22—C23	−1.0 (3)
C13—C1—C2—C7	0.6 (3)	C26—C25—C24—C23	−1.4 (4)
C13—C1—C2—C3	179.1 (3)	C18—C19—C20—C21	−179.5 (3)
C13—C9—C8—C7	−4.4 (3)	C20—C21—C22—C23	178.3 (3)
C13—C9—C10—C11	0.8 (3)	C21—C22—C23—C24	0.7 (4)
C13—C12—C11—C10	1.5 (3)	C25—C24—C23—C22	0.5 (4)
C8—C7—C2—C1	−1.4 (3)		
